# Author Correction: Immunogenicity and efficacy of fourth BNT162b2 and mRNA1273 COVID-19 vaccine doses; three months follow-up

**DOI:** 10.1038/s41467-023-37338-7

**Published:** 2023-03-22

**Authors:** Michal Canetti, Noam Barda, Mayan Gilboa, Victoria Indenbaum, Michal Mandelboim, Tal Gonen, Keren Asraf, Yael Weiss-Ottolenghi, Sharon Amit, Ram Doolman, Ella Mendelson, Dror Harats, Laurence S. Freedman, Yitshak Kreiss, Yaniv Lustig, Gili Regev-Yochay

**Affiliations:** 1 The Infection Prevention & Control Unit, Sheba Medical Center, Tel Hashomer, Ramat Gan, Israel; 2 Sackler School of Medicine, Tel-Aviv University, Tel Aviv, Israel; 3 ARC Innovation Center, Sheba Medical Center, Tel Hashomer, Ramat Gan, Israel; 4 Software and Information Systems Engineering, Ben-Gurion University of the Negev, Be’er Sheva, Israel; 5 Department of Biomedical Informatics, Harvard Medical School, Boston, MA USA; 6 Central Virology Laboratory, Public Health Services, Ministry of Health, Tel-Hashomer, Ramat Gan, Israel; 7 The Dworman Automated-Mega Laboratory, Sheba Medical Center, Tel-Hashomer, Ramat-Gan, Israel; 8 Clinical Microbiology, Sheba Medical Center, Tel Hashomer, Ramat Gan, Israel; 9 General Management, Sheba Medical Center, Tel Hashomer, Ramat Gan, Israel; 10 Biostatistics and Biomathematics Unit, Gertner Institute of Epidemiology and Health Policy Research, Sheba Medical Center, Tel Hashomer, Israel; 11 Vaccine Research Center, National Institute of Allergy and Infectious Diseases, National Institutes of Health, Bethesda, MD USA

**Keywords:** Clinical trials, SARS-CoV-2, RNA vaccines, Viral infection

Correction to: *Nature Communications* 10.1038/s41467-022-35480-2, published online 13 December 2022

The published version of the article contained an error in Figure 2, where the incorrect panel d had been included. This has been corrected in the HTML and PDF versions.

